# Mortality in Children with Optic Pathway Glioma Treated with Up-Front BB-SFOP Chemotherapy

**DOI:** 10.1371/journal.pone.0127676

**Published:** 2015-06-22

**Authors:** Josué Rakotonjanahary, Emilie De Carli, Matthieu Delion, Chantal Kalifa, Jacques Grill, François Doz, Pierre Leblond, Anne-Isabelle Bertozzi, Xavier Rialland, Brain Tumor Committee of SFCE

**Affiliations:** 1 Department of Pediatric Oncology, University Hospital, Angers, France; 2 INSERM CIE5 Robert Debre Hospital, Assistance Publique-Hôpitaux de Paris, University Paris Diderot, Sorbonne Paris Cité, Paris, France; 3 Department of Neurosurgery, University Hospital, Angers, France; 4 Department of Pediatric and Adolescent Oncology, Gustave Roussy Institute, Villejuif, France; 5 Department of Pediatric Oncology, Curie Institute and University Paris Descartes, Sorbonne Paris Cité, Paris, France; 6 Department of Pediatric Oncology, Oscar Lambret Center, Lille, France; 7 Department of Pediatric Oncology, University Hospital, Toulouse, France; University Hospital of Navarra, SPAIN

## Abstract

**Background:**

In terms of overall survival (OS), limited data are available for the very long-term outcomes of children treated for optic pathway glioma (OPG) with up-front chemotherapy. Therefore, we undertook this study with the aim of clarifying long-term OS and causes of death in these patients.

**Methods:**

We initiated and analyzed a historical cohort study of 180 children with OPG treated in France with BB-SFOP chemotherapy between 1990 and 2004. The survival distributions were estimated using Kaplan-Meier method. The effect of potential risk factors on the risk of death was described using Cox regression analysis.

**Results:**

The OS was 95% [95% CI: 90.6-97.3] 5 years after diagnosis and significantly decreased over time without ever stabilizing: 91.6% at 10 years [95% CI: 86.5-94.8], 80.7% at 15 years [95% CI: 72.7-86.8] and 75.5% [95% CI: 65.6-83] at 18 years. Tumor progression was the most common cause of death (65%). Age and intracranial hypertension at diagnosis were significantly associated with a worse prognosis. Risk of death was increased by 3.1[95% CI: 1.5-6.2] (p=0.002) for patients less than 1 year old at diagnosis and by 5.2[95% CI: 1.5-17.6] (p=0.007) for patients with initial intracranial hypertension. Boys without diencephalic syndrome had a better prognosis (HR: 0.3 [95% CI: 0.1-0.8], p=0.007).

**Conclusions:**

This study shows that i) in children with OPG, OS is not as favorable as previously described and ii) patients can be classified into 2 groups depending on risk factors (age, intracranial hypertension, sex and diencephalic syndrome) with an OS rate of 50.4% at 18 years [95% CI: 31.4-66.6] in children with the worst prognosis. These findings could justify, depending on the initial risk, a different therapeutic approach to this tumor with more aggressive treatment (especially chemotherapy) in patients with high risk factors.

## Introduction

Optic pathway gliomas (OPGs) account for approximately 5% of all tumor cerebri in children [1], and in approximately 30% of cases, they are associated with neurofibromatosis type 1 (NF1) [2–3]. Although a large majority of these gliomas are pilocytic astrocytomas [3], their evolution is unpredictable, with a 5-year overall survival (OS) rate of more than 90% [4–5] but a lower progression-free survival rate of 40% [6]. Because of their characteristics, they seem to constitute a specific entity among low-grade gliomas (LGGs): indeed, they expose affected patients to neurological, visual and endocrinal risks, and unlike other LGGs in which surgery is often the only course of treatment (for example, posterior fossa), their location does not allow total resection. In addition, recent biological studies suggest different molecular alterations between posterior fossa pilocytic astrocytomas and pilocytic astrocytomas in visual pathways [7].

Currently, the optimal treatment of OPGs remains extremely controversial. The possibility of stabilization, even with a spontaneous regression of lesions, does not seem to justify a systematic treatment of all patients with optic glioma [8]. When the tumor is limited to one optic nerve with loss of vision on that side, surgery is a possible therapeutic strategy [9]. However, the benefit is very questionable when the tumor involves the optic chiasm: partial surgery is possible but with the risk of inducing vision loss and/or hypothalamic disorders. Thus, for a long time, radiotherapy has been the standard treatment, with a dose of 45 Grays or more allowing stabilization of visual disorders and tumor growth [10]. However, this can concomitantly lead to second tumors and significant sequelae, particularly in terms of cerebral vascular radiation [11], dysfunction of hypothalamic-pituitary function [12] and/or slowing of cognitive development [6]. These effects are even more marked as treatment is administered in young children. For these reasons, and to maintain maximum future functions for these children, the French Society of Pediatric Oncology (SFOP) developed, from 1990, a BB-SFOP chemotherapy regimen (Baby Brain-French Society of Pediatric Oncology) that is specifically designed for children under 5 years of age [13]. This treatment included a combination of 6 antimitotics that are administered sequentially for 16 months and include carboplatin, procarbazine, etoposide, cisplatin, vincristine and cyclophosphamide. This chemotherapy was mainly intended to delay or even to avoid irradiation of the tumor: indeed, in cases of secondary progression, second-line chemotherapy or radiation therapy could be proposed depending on the age of the child. Beginning in 1995, this therapeutic strategy was extended to children over 5 years of age, including those carrying NF1, given the deleterious effects of radiotherapy in this type of disorder. Concurrently, other pediatric oncology groups [14–17] have developed a similar therapeutic approach that favors chemotherapy as a first-line treatment for such patients.

At present, these regimens, including first-line chemotherapy, have become the standard for the treatment of OPGs. However, although this therapeutic strategy has been applied for more than 20 years, most studies published in the literature do not consider the outcomes of children beyond 5 or 10 years of age [5,18–19]. These findings are even more surprising given that this tumor is well known for its slow evolution. For this reason, we chose to initiate a large historical cohort study of children treated with BB-SFOP as first-line chemotherapy, which represents a relatively homogeneous population to allow reliable comparisons between patients. These children were subjected to a very long-term follow-up to better define their outcomes. In the present study, we focused on mortality in these patients, with the aim of clarifying overall survival and answering three questions: is the OS of these patients as good as is generally described? What are the main causes of death and the main factors involved in the survival rate?

## Patients and Methods

### Patients

This is a historical cohort analysis of children less than 16 years of age who were treated in France for an OPG between June 1990 and December 2004. Using the French data base BB-SFOP (which lists all of the children treated in France with BB-SFOP chemotherapy, regardless of the type of brain tumor) and records from each French pediatric oncology center, we initiated a historical cohort study of patients who were required to meet the two following conditions: i) under 16 years of age at diagnosis; ii) had been treated with up-front chemotherapy according to the BB-SFOP protocol. Partial surgery or shunting was possible before the initiation of chemotherapy. Children who initially underwent gross-total or sub-total resection as well as those who initially received radiotherapy or chemotherapy that differed from the BB-SFOP regimen were excluded from the study.

Histologic confirmation of LGG was not mandatory when the clinical and radiographic characteristics were consistent with the LGG and biopsy was deemed unsafe or unnecessary (especially in NF1 patients). For each patient, we reviewed clinical data from the time of diagnosis to the date of the latest contact, including all of the treatments (chemotherapy, surgery, radiotherapy) applied during the evolution. The cause of death was that provided in the patient's record as stated by the clinician.

All of the parents or legal guardians of the patients signed informed consent forms before the initiation of BB-SFOP chemotherapy. Anonymized clinical data were collected and analyzed with the approval of the institutional review board (Comité de Protection des Personnes—Ouest 3, Poitiers).

### Chemotherapy Regimen

The specific regimen of chemotherapy applied herein has been described previously [13]. It consisted of three courses (A, B, and C) of six different drugs administered in seven three-course cycles. The planned duration of chemotherapy was 16 months. During course A, patients received carboplatin on day 1 and procarbazine on days 1 to 7. For course B, patients were administered etoposide and cisplatin on days 22 and 23, and for course C, patients received vincristine and cyclophosphamide on day 43. The next cycle was started on day 64.

### Statistical analysis

Data are presented as medians and ranges and as percentages. Events for OS were defined as the time from diagnosis to the date of death regardless of the cause. Data were collected for all patients, including those who were deceased. The cause of death was described as it was stated by the clinician in the patient's records. Living patients were censored at the date of the last follow-up or last contact. Patients included in this study were monitored from the time of diagnosis to March 30, 2014. The median follow-up time was computed for living patients. Survival distributions were estimated using the Kaplan-Meier method and compared among groups according to the presence or absence of risk factors. The log-rank test was used to test differences between these distributions. Cox univariate and multivariate analyses were used to calculate the hazard ratio (HR) and to estimate the effect of the potential risk factors on the risk of death. Graphical methods and time-interaction models were used to check the proportional hazards assumption for all potential risk factors. The candidate risk factors were as follows: age at diagnosis (< 1 year), sex, intracranial hypertension at diagnosis, diencephalic syndrome (DS), NF1 status, subsequent chemotherapy and radiotherapy and initial partial surgery. This selection was based on an analysis of the literature (age, sex, NF1 status, diencephalic syndrome) or on the hypothesis that initial intracranial pressure was likely to worsen the long-term prognosis. Concomitantly, we wanted to assess the influence of other treatments (surgery, radiotherapy, other chemotherapeutic regimens) applied after BB-SFOP chemotherapy on the long-term overall survival. Because gender has been previously described in the literature as a disease modifier [20], we decided in advance to use statistical interaction terms to evaluate the potential modification of OS by gender with age at diagnosis, gender with NF1 status, and gender with DS at diagnosis. For the Cox multivariate analysis, a step-down variable selection using Akaike’s information criterion was used as a stopping rule. All of the statistical tests were performed with a two-sided level of significance of 0.05. The statistical analysis was performed using Stata software 12.1.

## Results

### Patient characteristics

Between June 1990 and December 2004, a total of 445 children in France were treated with BB-SFOP chemotherapy. There were various indications (this chemotherapy can be applied in young children for brain tumors other than OPGs), and only 182 patients aged less than 16 years were identified as having been treated for OPG, using BB-SFOP chemotherapy as the first-line treatment. Two patients could not be included in the analysis because too much diagnosis data were missing from their records. Finally, our series included 180 children with a median follow-up of 13.6 years (range: 6.1–23.6). The main characteristics of these patients at the time of diagnosis are summarized in Table 1. Among the 180 patients, 79 had a follow-up ≥ 15 years, and 49 had a follow-up ≥ 18 years. Six patients were lost to follow-up (median follow-up: 11.8 years, range: 6.1–14.6).

**Table 1:** Characteristics of patients at diagnosis.

Characteristics	All patients	Total death
	**n = 180**	**n = 31**
Sex		
male	84 (46.6%)	10 (32.3%)
female	96 (53.4%)	21 (67.7%)
sex ratio (M/F)	0.87	0.47
Age (years)		
< 1	45 (25%)	15 (48.4%)
1–4	102 (56.7%)	11 (35.5%)
5–9	24 (13.3%)	2 (6.4%)
≥ 10	9 (5%)	3 (9.7%)
Median (IQR)	2.4 (1–4.2)	1 (0.5–3.6)
Year of diagnosis		
1990–2000	99 (55%)	20 (64.5%)
> 2000	81 (45%)	11 (35.5%)
NF1		
yes	60 (33.3%)	9 (29%)
no	120 (66.7%)	22 (71%)
Diencephalic syndrome ^1^		
yes	32 (17.8%)	11 (35.5%)
no	148 (82.2%)	20 (64.5%)
Intracranial Hypertension ^2^		
yes	48 (26.7%)	11 (35.5%)
no	132 (73.3%)	20 (64.5%)

^1^ Diencephalic syndrome was defined as failure to thrive and a diagnostic weight between the 2^nd^ and 5^th^ percentile.

^2^ Intracranial hypertension was defined by clinical symptoms associated with radiological hydrocephalus.

Fifteen patients with diencephalic syndrome (DS) were less than 1 year old, 12 were between 1 and 2 years old, and 5 were more than 2 years old. Fifteen patients presented with the spread of disease at diagnosis, including 6 patients with DS. However, spinal axis MRI was not systematically performed, and some metastases may not have been diagnosed.

In terms of diagnosis, 109 of 180 patients (60.5%) underwent surgery (biopsies and/or partial resections) initially or during the course of treatment. Among the 71 patients without histological diagnosis, 46 were NF1 patients, and 25 were non-NF1 patients: for these patients, the clinical data did not specify the reasons for not performing surgical biopsy and/or partial resection. When a histological diagnosis was available, it confirmed LGG in 104 patients and showed grade II or III astrocytoma in 5 patients; however, unfortunately, a pathology review could not be performed. For the other children, MRI imaging was considered sufficient to establish a diagnosis of OPG.

### Treatments

In terms of treatment, it was decided to undertake chemotherapy when there was evidence of clinical progression (especially significant deterioration of vision or neurological signs) and/or radiological tumor progression. The median time between diagnosis and the first course of BB-SFOP was 21 days (interquartile range: 11–71 days). One hundred and twenty-six patients (70%) received a total of 7 cycles of chemotherapy that were initially planned in the treatment scheme, 7 (3.9%) received more than 7 cycles because of a persistent response to chemotherapy at the end of the planned scheme, and 47 (26.1%) received fewer than 7 cycles. In most cases, premature discontinuation of chemotherapy was due to tumor progression (48%) or a lack of response (21%) to treatment, and other discontinuations were due to toxicity (10%), death (6%), or parental refusal to continue the treatment (4%). In 5 cases, the clinical data did not specify the reasons for stopping BB-SFOP chemotherapy prematurely.

The treatments applied after BB-SFOP are summarized in S1 Table: approximately 46% of the patients (83 of 180) did not receive any other chemotherapy, while 54% (97 of 180) received between 1 and 8 additional lines of chemotherapy. Ninety-five of the 180 patients (52.7%) received no other specific treatment apart from chemotherapy. Radiotherapy alone was performed in 28/180 patients (15.5%), surgery alone (partial excision) in 30/180 patients (16.8%) and surgery associated with radiotherapy in 27/180 patients (14.9%). Radiotherapy was performed in 7 patients with NF1. Age at radiotherapy varied from 2.7 to 21 years old (median: 8 years). A total of 69 children (38.3%) required a shunt and/or shunt revision during the management of the disease. Fifty-six patients (31.1%) did not receive any other specific treatment after BB-SFOP chemotherapy (additional lines of chemotherapy, surgery and/or radiotherapy).

### Overall Survival

**Fig 1:**
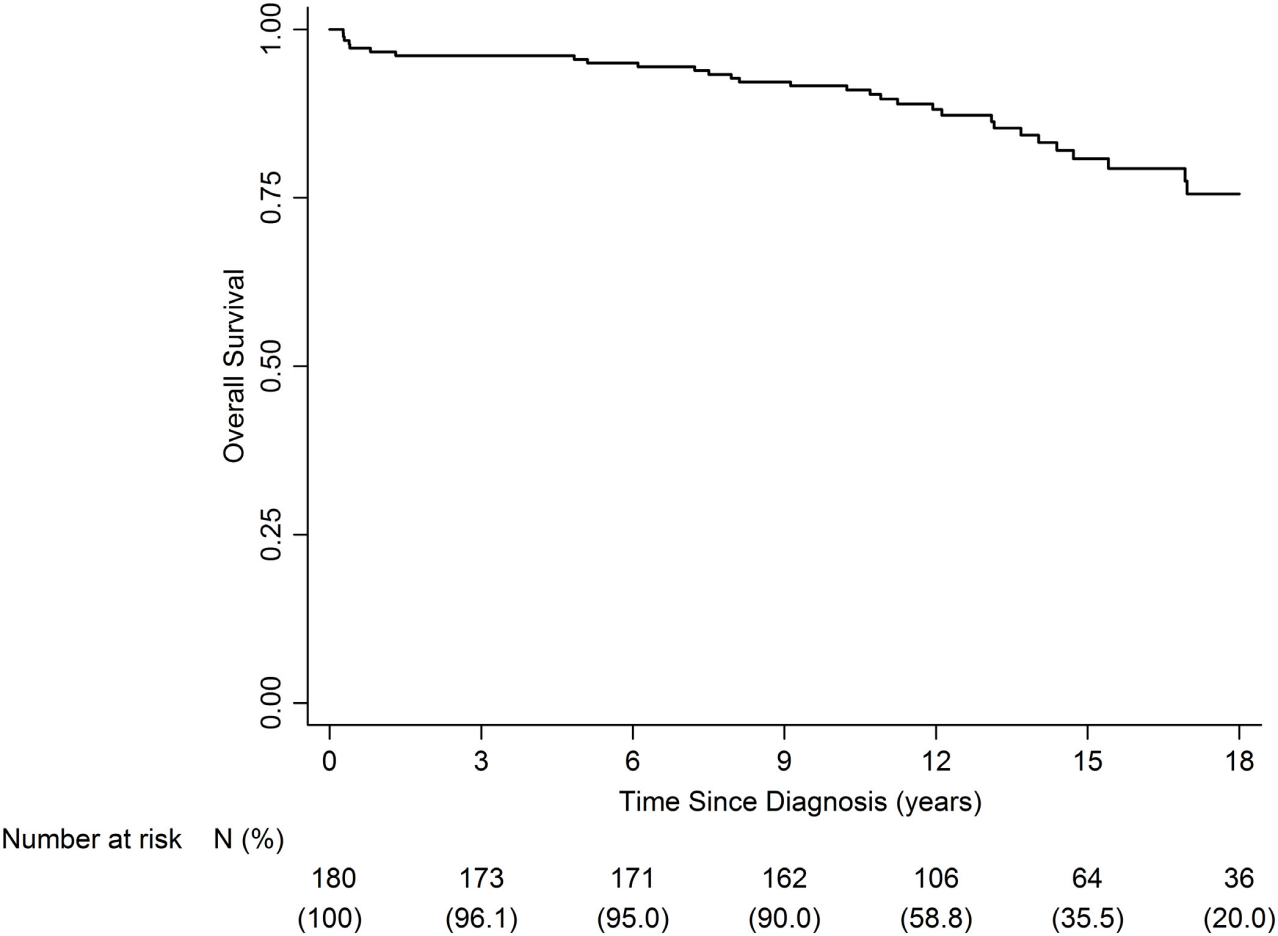
Overall Survival for the whole population with optic pathway glioma.

Computed from the date of diagnosis, the 5- and 10-year OS rate were 95% [95% CI: 90.6–97.3] and 91.6% [95% CI: 86.5–94.8], respectively, for the whole population. Among the 79 patients who could have a follow-up of ≥ 15 years, the OS rate at 15 years was 80.7% [95% CI: 72.7–86.8], and among the 49 patients who could have a follow-up of ≥ 18 years, the OS rate at 18 years was 75.5 [95% CI: 65.6–83] (Fig 1).

**Fig 2:**
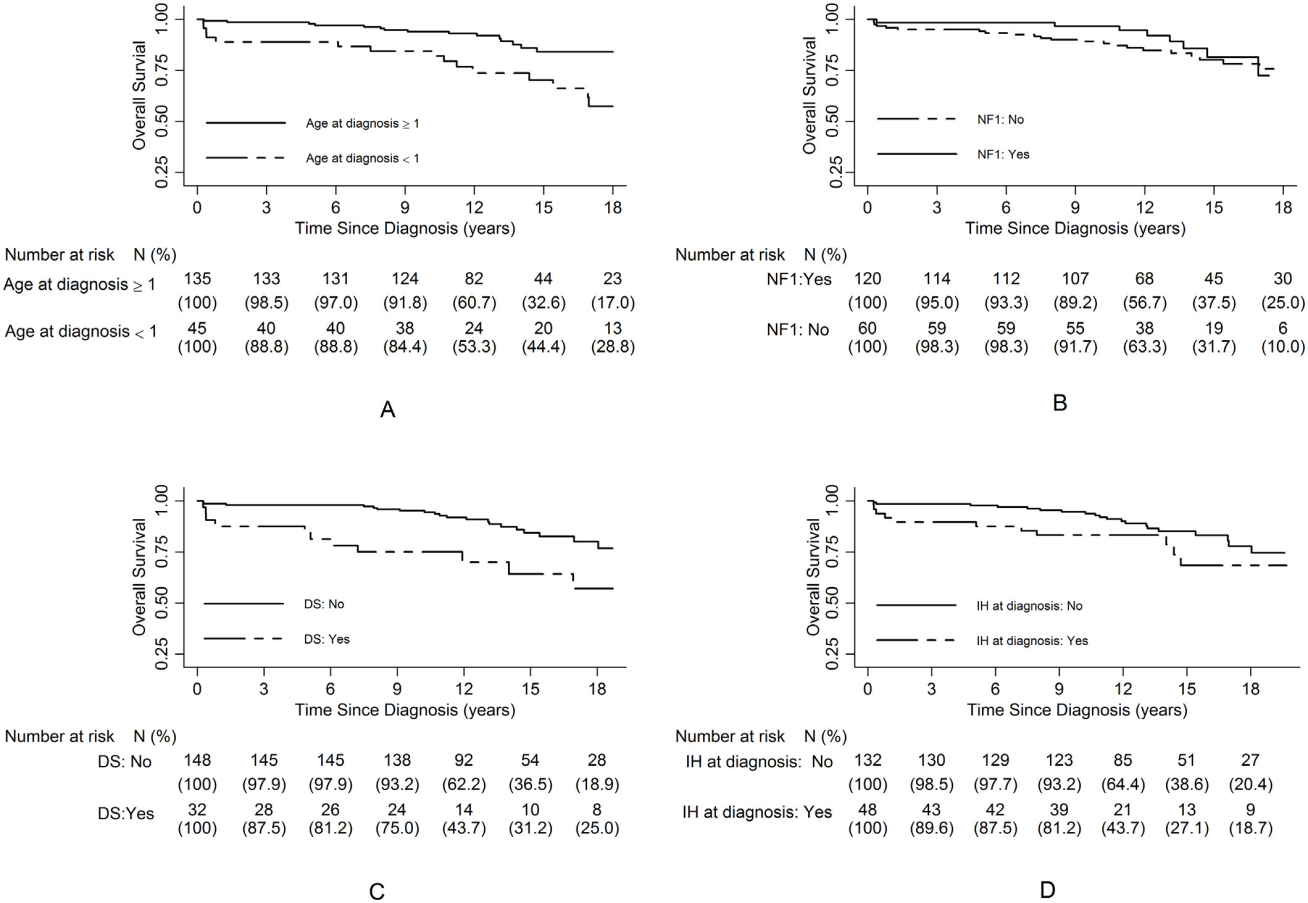
Overall survival according to age at diagnosis (A), NF1 status (B), presence of DS (C) or intracranial hypertension (IH) (D) at diagnosis.

We analyzed different clinical criteria that might influence the results: sex, age at diagnosis, NF1 associated with OPG, presence of DS or intracranial hypertension at diagnosis. The results are summarized in Fig 2.

According to the Cox univariate analysis and the Kaplan-Meier survival analysis (Table 2), males had a better survival than females, although this difference was not statistically significant (HR: 0.4 [95% CI: 0.2–1], p = 0.06). We observed a better prognosis in children with NF1 associated with OPG (HR: 0.8 [95% CI: 0.6–0.9], p = 0.035). Patients with intracranial hypertension at diagnosis also had a worse prognosis (HR: 4 [95% CI: 1.2–13.3], p = 0.023). The risk of death was increased in children less than 1 year of age (HR: 2.6 [95% CI: 1.3–5.4]), p = 0.006) and in those with DS at diagnosis (HR: 2.8 [95% CI: 1.3–6], p = 0.005). We also analyzed the potential influence of the applied treatments (initial partial surgery, chemotherapy and/or radiotherapy administered after initial BB-SFOP treatment). There was no statistically significant difference in the survival rates and the risk of death according to whether the patients were or were not eligible for initial partial resection (HR: 1.2 [95% CI: 0.5–2.7], p = 0.65), or had or had not received radiotherapy (HR: 0.9 [95% CI: 0.4–1.9]) (p = 0.88) or chemotherapy (HR: 1.04 [95% CI: 0.9–1.2]) (p = 0.56) after BB-SFOP. Survival rates according to treatments are shown in Table 2 and S1 Fig.

**Table 2:** Overall Survival distributions and Hazard Ratios depending on the clinical data at diagnosis and treatments (p values according to univariate Cox regression analysis).

	Length of follow-up (years)	Survival rate (%)	95% CI	Number of patients at risk	Survival rate (%)	95% CI	Number of patients at risk	HR (95% CI) p value
**OS for the whole population** (N = 180)								
	5	95.0	90.6–97.3	172	-	-	-	-
	10	91.6	86.5–94.8	153	-	-	-	
	15	80.7	72.7–86.8	64	-	-	-	
	18	75.5	65.6–83.0	36	-	-	-	
**Sex**		**Male**			**Female** *			0.4
								(0.2–1)
	5	95.2	87.8–98.2	81	93.7	86.6–97.1	91	
	10	92.8	84.8–96.7	74	90.5	82.5–94.9	79	
	15	86.5	74.6–93.1	33	75.7	63.5–84.3	31	0.06
	18	86.5	74.6–93.1	22	65.1	48.5–77.3	14	
**NF1 status**		**NF1**			**Non NF1** *			0.8
								(0.6–0.9)
	5	98.3	88.7–99.7	59	93.3	87.1–96.6	113	
	10	96.6	87.1–99.1	54	89.1	82.0–93.5	99	
	15	81.4	63.4–91.1	19	80.1	70.4–86.9	45	0.035
	18	72.4	46.0–87.4	6	75.7	64.3–83.9	30	
**Intracranial hypertension**		**Present**			**Absent** *			4
								(1.2–13.3)
	5	87.5	74.2–94.1	43	96.9	92.1–98.8	129	
	10	83.2	69.2–91.2	37	94.6	89.1–97.4	116	
	15	68.4	47.4–82.4	13	85.1	76.5–90.7	51	0.023
	18	68.4	47.4–82.4	9	77.7	65.4–86.1	27	
**Age at diagnosis**		**< 1 year**			**≥ 1 year** *			2.6
								(1.3–5.4)
	5	86.6	72.7–93.7	40	97.0	92.3–98.8	132	
	10	84.4	70.1–92.2	36	94.0	88.3–96.9	117	
	15	61.4	53.1–82.1	20	84.0	74.0–90.4	44	0.006
	18	54.6	37.9–72.7	13	84.0	74.0–90.4	23	
**Diencephalic syndrome**		**Present**			**Absent** *			2.8
								(1.3–6)
	5	81.2	62.9–91.1	27	97.9	92.8–99.3	145	
	10	75.0	56.2–86.6	23	95.2	90.2–97.6	130	
	15	64.1	42.1–79.6	10	84.3	75.4–90.2	54	0.005
	18	57.0	33.4–74.9	8	80.0	69.1–87.4	28	
**Initial partial surgery**		**Yes**			**No** *			1.2
								(0.5–2.7)
	5	94.7	80.5–98.6	37	94.4	89.0–97.1	135	
	10	92.1	77.5–97.4	34	91.5	85.5–95.1	119	
	15	76.3	55.5–88.3	16	81.9	72.7–88.3	48	0.657
	18	70.4	47.6–84.7	12	77.3	65.7–85.3	24	
**Subsequent chemotherapy**		**Yes**			**No** *			1
								(0.9–1.2)
	5	94.8	88.1–97.8	93	95.2	87.7–98.2	79	
	10	89.6	81.6–94.3	80	94.0	86.1–97.4	73	
	15	76.3	64.2–84.7	30	85.8	73.6–92.6	34	0.560
	18	67.7	51.1–79.8	16	79.3	64.0–88.7	20	
**Subsequent radiotherapy**		**Yes**			**No** *			0.9
								(0.4–1.9)
	5	98.2	87.8–99.7	55	92.8	86.6–96.2	117	
	10	90.8	79.4–96.1	49	91.9	85.6–95.6	104	
	15	79.3	64.7–88.4	30	81.4	70.4–88.6	34	0.881
	18	75.7	59.6–86.1	19	74.6	59.8–84.6	17	

* Reference for HR

In the Cox multivariate analysis (Table 3), an age at diagnosis of less than 1 year (HR: 3.1 [95% CI: 1.5–6.2], p = 0.002) and the presence of initial intracranial hypertension (HR: 5.2 [95% CI: 1.5–17.6], p = 0.007) were associated with a worse OS. Neither NF1 status nor the presence of DS at diagnosis appeared to be discriminant prognostic factors. When introducing an interaction term between the group of patients with DS and the variable sex, the association between the DS group and OS varied by gender. Boys without DS had a better prognosis compared with girls with or without DS and boys with DS (HR: 0.3 [95%CI: 0.1–0.8], p = 0.007).

**Table 3:** Multivariate Cox regression analysis of factors associated with Overall Survival in patients with OPG.

Risk Factors *	No. Of Patients	Hazard Ratio	95% CI	p
**Age at Diagnosis < 1 year**	45	3.1	1.5–6.2	0.002
**Intracranial Hypertension at Diagnosis**	48	5.2	1.5–17.6	0.007
**Boys without Diencephalic Syndrome**	70	0.3	0.1–0.8	0.007

* The candidate risk factors were: age at diagnosis (< 1 year), sex, intracranial hypertension at diagnosis, diencephalic syndrome, NF1 status, and subsequent treatment (chemotherapy, radiotherapy and initial partial surgery). Interaction terms used: gender with age at diagnosis, gender with NF1 status, gender with DS at diagnosis.

**Fig 3:**
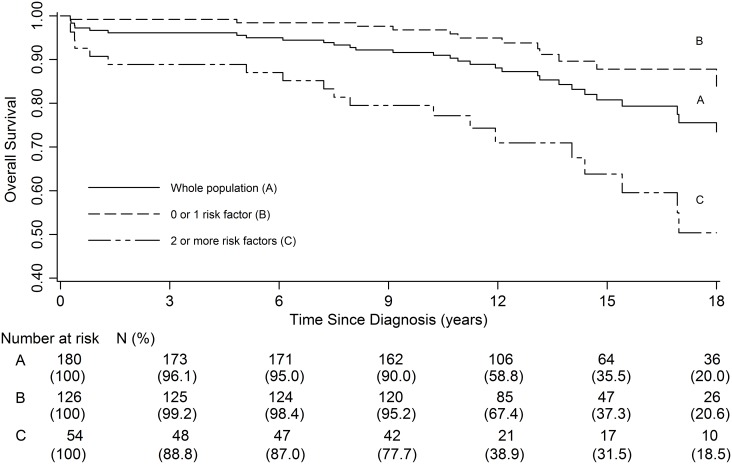
Overall survival according to the number of risk factors present at diagnosis: whole population (A), 0 or 1 risk factor (B), 2 or more risk factors (C).

Using these factors associated with a poor prognosis (age, intracranial hypertension, sex and DS), we were able to describe 2 groups of patients: B (absence or presence of one of these poor prognosis factors); C (presence of 2 or more of these factors). There was a statistically significant difference in the 5, 10, 15 and 18-year OS between group B and C (HR: 4.5 [2.1–9.2], p<10^−3^). The OS rate at 18 years was 87.8% in the first group [95%CI: 78.4–93.3] compared to 50.4% in the second group [95%CI: 31.4–66.6]. These results are summarized in Fig 3.

### Deceased patients

The main data concerning deceased patients are summarized in S2–S4 Tables. A total of 31 of 180 patients (17.2%) died: 10 patients died less than 6 years after diagnosis (group 1), 11 between 6 and 12 years (group 2), and 10 more than 12 years after diagnosis (group 3). Death was essentially due to tumor progression, as defined according to the RECIST (Response Evaluation Criteria in Solid Tumors) [21] (65%), but 5 patients (16%) died due to a second tumor (1 in groups 1 and 2, and 3 in group 3), 4 (13%) of vascular complications—thrombosis or cerebral hemorrhage outside the tumor–(1 in group 1, 3 in group 3), and 2 (6%) of chemotherapy toxicity (2 in group 1). A second tumor was associated with NF1 in 3/5 cases. Patients who developed a second tumor received BB-SFOP chemotherapy only in 1 case, 1 additional line of chemotherapy (vincristine-carboplatin) in 3 cases and 2 additional lines of chemotherapy in 1 case (vinblastine weekly for 14 months followed by temozolomide for 1 month). Only 1/5 patients received radiotherapy. A second tumor developed in the central nervous system in 4/5 cases: a high-grade glioma located in the brainstem (2 cases), in the frontal area (1 case) and in the temporo-parietal area (1 case). In 1 case (a patient with NF1), the second tumor was a sarcoma that had developed outside the central nervous system. Among the 4 patients with vascular complications, only 1 received radiotherapy. Among the deceased patients, 9 of 31 (29%) had NF1 associated with OPG, and these NF1 patients mostly died long after diagnosis (6/9 in group 3) from tumor progression (5/9) or a second tumor (3/9).

Among 48 patients who presented with intracranial hypertension at diagnosis, 11 died (22.9%): 6 died before the 6 years of follow-up, 2 between 6 and 12 years of follow-up and 3 after 12 years of follow-up. Among the 32 patients with DS at diagnosis, 11/32 (34.3%) died: 6 and 5 patients died less and more than 6 years after diagnosis, respectively (3 in group 2 and 2 in group 3). These 11 children had a median age of 0.7 years at diagnosis (interquartile range: 0.4–1.1 years) compared with 1 year of age for all patients with DS (interquartile range: 0.5–3.6 years), and 3/11 suffered from disseminated disease at diagnosis. Among all of the patients with the spread of disease at diagnosis, 5 died from tumor progression at 5 months, 10 months, 7 years, 8 years and 11 years after diagnosis.

Among the 31 deceased patients, 7 (22.5%) did not have a histological diagnosis, while 24 others were carrying low-grade tumors without evidence of malignancy. When comparing patients with and without a histological diagnosis, we did not detect significant differences in the 15-year OS between the 2 groups: 77% [95% CI: 66.1–85] and 87% [95% CI: 73.7–93.8] (p = 0.15). Survival rates according to histology are shown in S2 Fig.

## Discussion

To our knowledge, this is one of the largest series ever published describing children who were diagnosed with an OPG and initially treated uniformly using the same chemotherapeutic regimen with a long-term follow-up.

OPGs are known to have a slow but unpredictable course, and although rapid progression can be observed in some patients, most authors indicate a very good 5-year OS for this tumor, varying between 92% and 98% [5,18–19]. After 5 years of follow-up, our study revealed comparable results to these series. However, because we extended the follow-up beyond 5 years, we observed that after this period, survival significantly decreased over time without ever stabilizing. After 15 years, the OS rate was 80.7%, and after 18 years, it was only 75.5%. A number of patients died as a result of tumor progression more than 10 years after diagnosis, which is probably due to the very slow evolution of this type of glioma. Therefore, based on our data, we think that survival, when evaluated 5 or 10 years after diagnosis, may not be representative of the true survival experience of children with OPG because of the characteristics of this tumor. Consequently, there is a need to study survival over a longer period.

The role of NF1-associated OPG remains controversial. While some authors did not find a significant difference between progression-free survival (PFS) rates in children with or without NF1 [15–22], Fouladi et al. [23], in 2003, discovered a 6-year OS rate of 96% in NF1 patients compared with 80% in non-NF1 patients, but this series included patients with active treatment immediately after diagnosis and patients without initial therapeutic intervention. More recently, Gnekow et al, in a series of 415 supratentorial midline LGG patients [24], did not find any differences in OS in NF1 compared with non-NF1 patients, but these results included all localizations of LGG. In our series, we did not detect statistically significant differences between NF1 and non-NF1 patients in the Cox multivariate analysis when considering NF1 as predictive factor in the survival rate. Despite a trend toward a better prognosis in NF1 patients during the first years of follow-up, this difference completely disappeared over time with the same OS rate between the 2 groups at the 15-year follow-up. A majority of deaths in NF1 patients occurred after the 10-year follow-up and following tumor progression, which suggested that these children could die later than the non-NF1 patients. They did not die of other phakomatosis complications but of an OPG, and the administered treatments, especially radiotherapy [25–26], did not seem to have an effect on this long-term evolution. However, these results could be influenced by the observation that the percentage of irradiated children, in our series as in others, was generally lower in NF1 than in non-NF1 patients. When considering our results and those in the literature, the slower progression might have been easier to initially control in these NF1 patients but was likely to worsen over time. This finding again appears to highlight the need for the sufficient follow-up of these tumors to accurately assess the outcomes of the children.

Age is another prognostic factor noted by many authors: in numerous series, the analysis of pediatric patients with an OPG has demonstrated that an age of less than 1 year is an independent negative prognostic factor [5, 19, 23–24, 27–28]. These results are very similar to ours. The difference observed in OS was statistically significant at the first 5-year follow-up and did not change thereafter. In other words, with the knowledge that the deaths were mostly due to tumor progression, the risk of treatment failure appeared to be higher for children less than 1 year old than for older children and did not improve over time.

Although it is a rare disorder, the presence of a DS at diagnosis is a third prognostic factor noted in the literature, especially when it is associated with leptomeningeal spread [13, 24, 29]. In our series, the number of children with a spread of disease at diagnosis was too small to draw any conclusions concerning the association between DS and disseminated disease. Recently, Kilday et al. [30] reported a series of 9 children with DS who were treated with up-front chemotherapy followed, in cases of progression, by several lines of chemotherapy and/or surgery without radiotherapy: these patients demonstrated a 5-year OS of 100%, but the conclusions appeared to be difficult to determine because the number of patients was very small and the follow-up was short. Our study results did not confirm these findings, and moreover, our conclusions might seem surprising given that DS and female appeared to be a poor prognostic factor. This result can be compared with the recent study reported by Fisher et al. [20], who found that gender may be a disease modifier in the development of clinically significant OPG in children with NF1. However, to our knowledge, no study specifically investigating very long-term OS in these children with DS has ever been published. The number of children with an OPG and DS in our cohort is one of the largest reported to date, and the distribution between males and females is similar. Because these children also had a very long follow-up (median: 13.6 years, range: 9.6–23.5), we believe that our results merit interest. Nevertheless, we do not have an explanation for the present findings, and we did not find any studies in the literature comparing boys and girls with an OPG and DS: a prospective study of these patients is likely needed to better understand the evolution of these two groups of children.

A few reports have been conducted to investigate intracranial hypertension in patients with an OPG [31–32], but none of them considered this symptomatology at diagnosis as a possible predictive factor of the patient outcomes. Thus, in our series, it was surprising to note a slightly significant difference in the OS rate with a worse prognosis in patients with these symptoms. Intracranial hypertension did not correlate with other factors. Half of the patients with intracranial hypertension died before 6 years of follow-up. The tumor characteristics (location, speed of development, leptomeningeal dissemination) could explain why but we were not able to demonstrate it. This question should likely be investigated in future studies.

There are several limitations of the present study that should be acknowledged. First, this is a historical cohort study in which only OS was analyzed without reference to event-free survival (EFS) or progression-free survival (PFS). However, the main purpose of this study was to assess the mortality of children with OPG who were treated with up-front chemotherapy. In addition, analyses of EFS and PFS would require a consensus regarding the definition of the clinical and radiological criteria used. At present, there are still too many variations of these criteria and of the tumor measurements, and discrepancies in response categorizations among readers have been well demonstrated in several studies [33–34]. Under these conditions, comparisons between different published series lack reliability. The second limitation is that this series included only children with initial symptoms that justified treatment. By definition, it excluded children with no or few symptomatic OPGs, in whom the condition might have remained stable over time, and thus, only monitoring was needed. Therefore, our findings relate only to this type of patient with initially progressive disease and cannot be considered as a prognostic evaluation of all OPGs. A third limitation of this study is the number of patients with a histological diagnosis. Most studies concerning optic pathway glioma have reported a histological diagnosis in a limited number of patients, and the diagnosis was usually established based on MRI. For example, in the study reported by Mishra et al. [5], 445 patients were identified through the SEER database as being diagnosed with an OPG, but histology was available in only 131 patients (29.5%). Thus, because there was no central review, we cannot definitively exclude that some of the tumors had been misclassified or that some patients were carrying tumors other than LGG. A fourth limitation was that the cause of death was noted by the clinician in the patient’s record, and in the present study, it was impossible to verify these data long after death. Therefore, we cannot exclude the possibility that, for some patients, the cause of death differed from that identified in the file. A fifth limitation was the analyzed prognostic factors. We limited our study to clinical criteria because a radiological review (in progress) would not be able to answer all of our questions, mainly because of differences in the techniques used, depending on the centers and the dates on which the MRIs were performed. In addition, the number of deceased patients in our series was not large enough to enable the detection of other risk factors. We can no longer take into account recent advances in the tumor biology of LGG [35–41]. Nevertheless, there is no doubt that, in the future, advances in pathology and molecular biology will greatly improve the understanding of LGG and, consequently, of OPGs. This progress will facilitate the design of new treatments that are better targeted and adapted to different types of tumors so that significantly improved results in terms of survival are realized in the future.

Nevertheless, the results of this study allowed us to draw three conclusions. First, the prognosis of OPG in children, in terms of OS, is probably not as favorable as previously described when patients have a follow-up that is long enough to account for later deaths (more than 10 years after diagnosis). If we want to properly assess the evolution of these children, it seems necessary not to limit the follow-up to 5 or 10 years but to extend it for a very long period of 15 years or more. Second, tumor progression appears to be the main cause of death, especially at time points longer than 6 years after diagnosis. Deaths related to vasculopathy or second tumors are much less frequent and do not appear to be systematically linked to NF1 status and/or radiotherapy. Third, taking into account the risk factors that we have identified, children with OPG appear to be classified into 2 groups, each with different issues. In the first group, which had a very good 18-year OS, the key question appears to concern long-term disease sequelae and/or treatment (especially visual outcomes and hypothalamic syndrome). In contrast, in the second group with poor long-term survival, the main problem appears to be the effectiveness of the treatments. Future advances in therapeutics resulting from a better understanding of tumor biology should focus on patients with a worse prognosis.

## Supporting Information

S1 Fig Overall survival according to subsequent chemotherapy (A), subsequent radiotherapy (B) and initial partial surgery (C). (TIF)

S2 Fig Overall survival according to histology. (TIF)

S1 Table Treatments applied after first-line BBSFOP chemotherapy and patient outcomes (surgical procedures for intracranial hypertension could be performed before, during or after BBSFOP). (DOC)

S2 Table Characteristics of dead patients depending on the time between diagnosis and death: Group 1. (DOC)

S3 Table Characteristics of dead patients depending on time between diagnosis and death: Group 2. (DOC)

S4 Table Characteristics of dead patients depending on time between diagnosis and death: Group 3. (DOC)
