# Supplementary figures and images for: Mortality in Children with Optic Pathway Glioma Treated with Up-Front BB-SFOP Chemotherapy
Source: PLoS One. 2015 Jun 22;10(6):e0127676. doi: 10.1371/journal.pone.0127676 (PMC4476571; doi:10.1371/journal.pone.0127676)

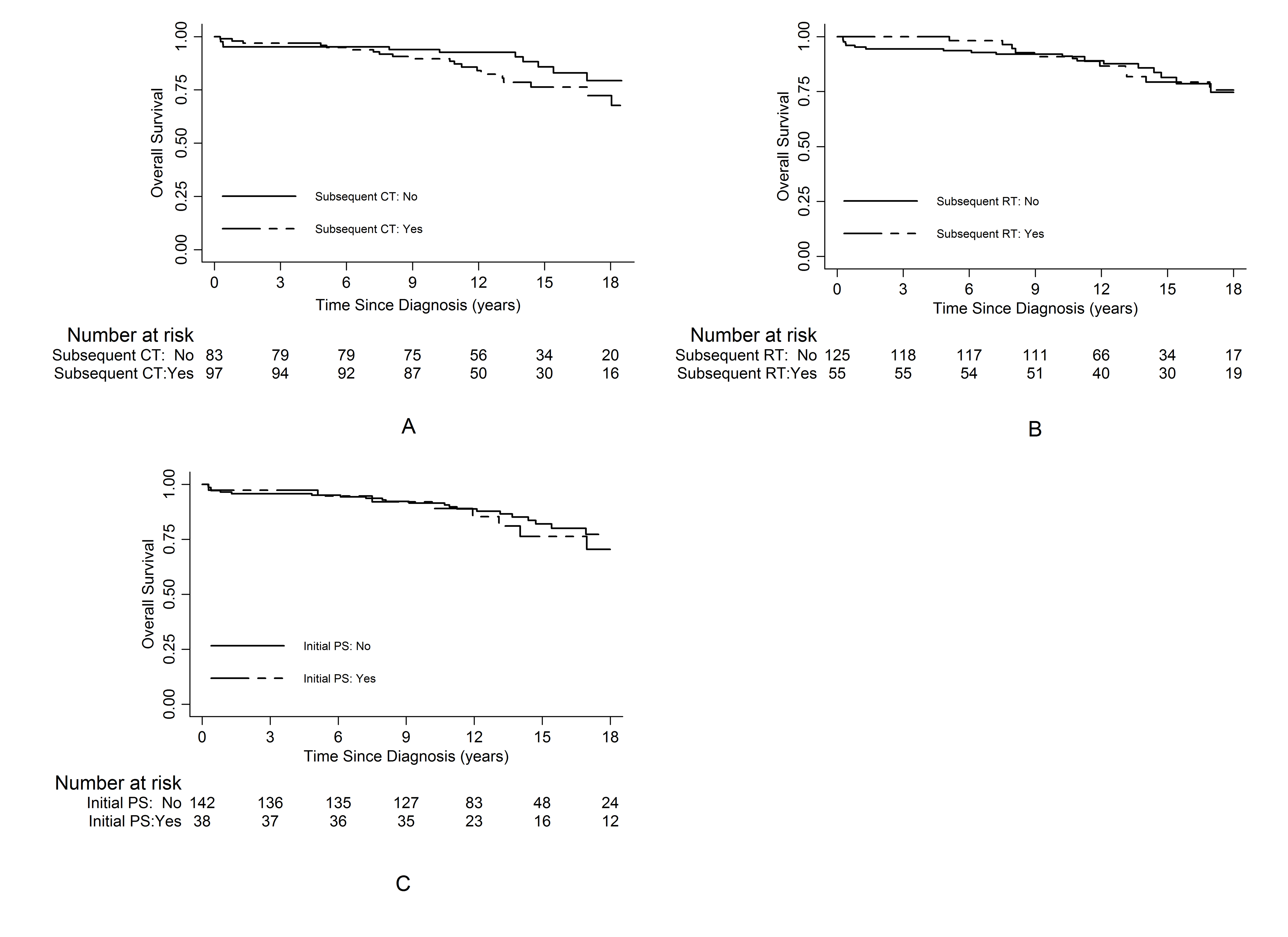

Supplement: S1 Fig — (TIF) [file pone.0127676.s001.tif]

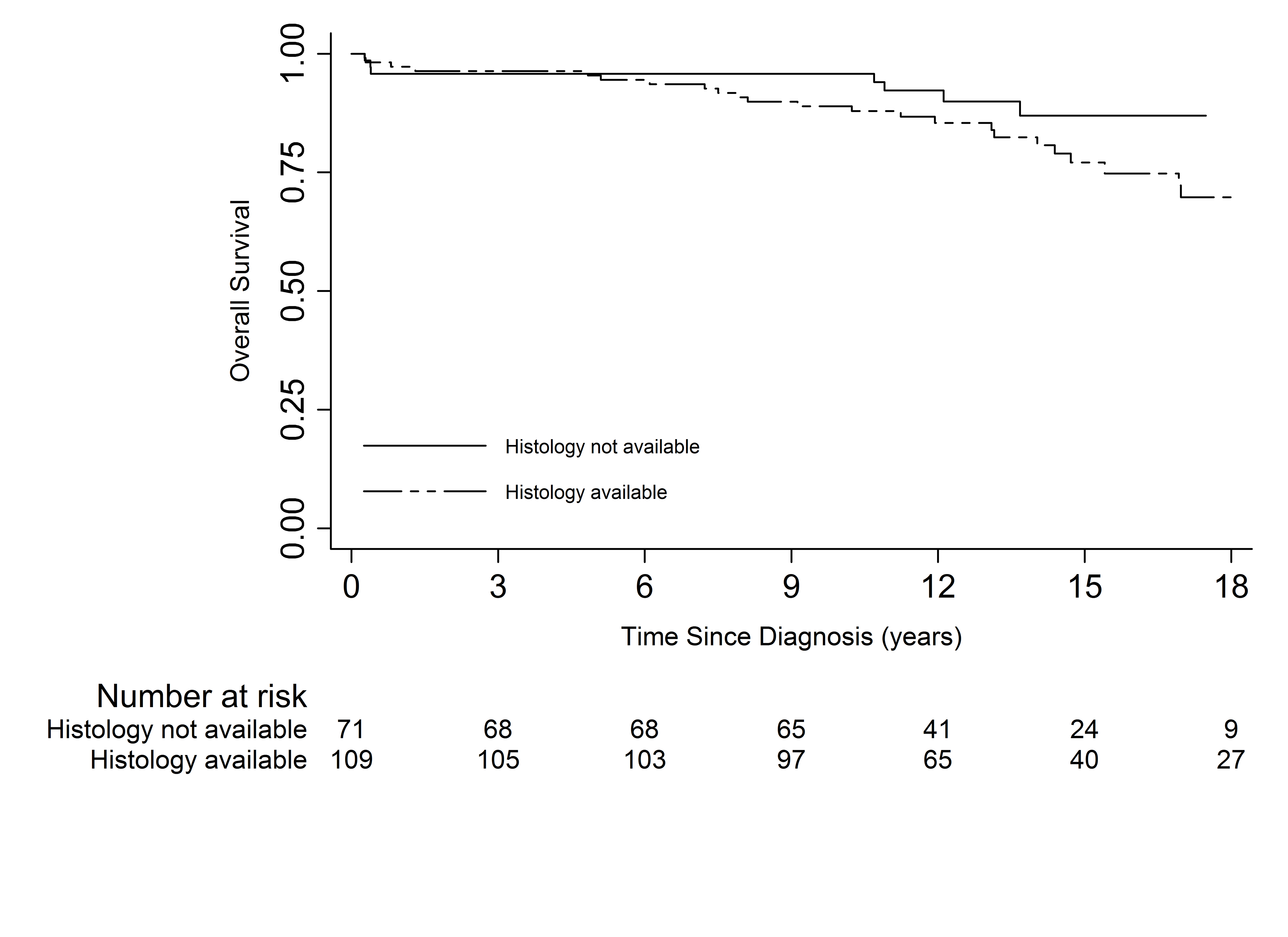

Supplement: S2 Fig — (TIF) [file pone.0127676.s002.tif]
