# Supplementary material for: Mortality in Children with Optic Pathway Glioma Treated with Up-Front BB-SFOP Chemotherapy
Source: PLoS One. 2015 Jun 22;10(6):e0127676. doi: 10.1371/journal.pone.0127676 (PMC4476571; doi:10.1371/journal.pone.0127676)
Supplement: S1 Table — (DOC) [file pone.0127676.s003.doc]

**S1 Table. Treatments applied after first-line BBSFOP chemotherapy and patient outcomes (surgical procedures for intracranial hypertension could be performed before, during or after BBSFOP)** (because the numbers were small, the percentages were approximations to the nearest tenth).

|  | |  | |  | | | | |  |  | |  | | | | |  |  | | | | |  | | | | | |  | |  | |  | |  | |  | |
| --- | --- | --- | --- | --- | --- | --- | --- | --- | --- | --- | --- | --- | --- | --- | --- | --- | --- | --- | --- | --- | --- | --- | --- | --- | --- | --- | --- | --- | --- | --- | --- | --- | --- | --- | --- | --- | --- | --- |
| **Status of patients** | Dead | | Alive | | | |  | Dead | | | Alive | | |  | | Dead | | | | | | Alive | | | | |  | Dead | | | | Alive | |  | | **Total patients** | |  |
|  | n=11 | | n=72 | | | |  | n=17 | | | n=64 | | |  | | n=2 | | | | | | n=10 | | | | |  | n=1 | | | | n=3 | |  | | **n=180** | |  |
|  |  | |  | | | |  |  | | |  | | |  | |  | | | | | |  | | | | |  |  | | | |  | |  | |  | |  |
|  |  | |  | | | |  |  | | |  | | |  | |  | | | | | |  | | | | |  |  | | | |  | |  | | | |  |
| **Number of lines of chemotherapy after BBSFOP** | 0 | | | | | |  | 1 to 3 | | | | | |  | | 4 to 5 | | | | | | | | | | |  | 6 or more | | | | | |  | | | |  |
|  |  | | | | | | | | | | | | | | | | | | | | | | | | | | | | | | | | | | | | | |
| **Number of patients** | **11 (6%)** | | **72 (40%)** | | | |  | **17 (9.5%)** | | | **64 (35.6%)** | | |  | | **2 (1.1%)** | | | | | | **10 (5.6%)** | | | | |  | **1 (0.5%)** | | | | **3 (1.7%)** | |  | | **180** | |  |
|  |  | | | | | | | | | | | | | |  | | | | | | | | | | | | | | | | | | | | | | |  |
|  |  | | | | | | | | | | | | | | | | | | | | | | | | | | | | | | | | | | | | | |
| **Number of patients with specific treatment different from chemotherapy** |  | | | | | | | | | | | | | | | | | | | | | | | | | | | | | | | | | | | | | |
|  |  | | | | | | | | | | | | | | | | | | | | | | | | | | | | | | | | | | | | | |
| - Surgery (partial resection) | 1 (0.5%) | | 9 (5%) | | | |  | 4 (2.2%) | | | 7 (3.9%) | | |  | | 1 (0.5%) | | | | | | 6 (3.5%) | | | | |  | 0 (0%) | | | | 2 (1.2%) | |  | | 30 (16.8%) | |  |
|  |  |  |  |  |  |  |  |  |  |  |  |  |  |  |  |  |  |  |  |  |  |  |  |  |  |  |  |  |  |  |  |  |  |  |  |  |  |  |
| - Radiotherapy | 2 (1.1%) | | 10 (5.6%) | | | |  | 3 (1.6%) | | | 12 (6.7%) | | |  | | 0 (0%) | | | | | | 1 (0.5%) | | | | |  | 0 (0%) | | | | 0 (0%) | |  | | 28 (15.5%) | |  |
|  |  |  |  |  |  |  |  |  |  |  |  |  |  |  |  |  |  |  |  |  |  |  |  |  |  |  |  |  |  |  |  |  |  |  |  |  |  |  |
| - Surgery ± Radiotherapy | 1 (0.5%) | | 4 (2.2%) | | | |  | 4 (2.2%) | | | 14 (7.8%) | | |  | | 1 (0.5%) | | | | | | 3 (1.6%) | | | | |  | 0 (0%) | | | | 0 (0%) | |  | | 27 (14.9%) | |  |
|  |  |  |  |  |  |  |  |  |  |  |  |  |  |  |  |  |  |  |  |  |  |  |  |  |  |  |  |  |  |  |  |  |  |  |  |  |  |  |
| - No specific treatment different from chemotherapy | 7 (3.9%) | | 49 (27.2%) | | | |  | 6 (3.5%) | | | 31 (17.2%) | | |  | | 0 (0%) | | | | | | 0 (0%) | | | | |  | 1 (0.5%) | | | | 1 (0.5%) | |  | | 95 (52.8%) | |  |
|  |  |  |  |  |  |  |  |  |  |  |  |  |  |  |  |  |  |  |  |  |  |  |  |  |  |  |  |  |  |  |  |  |  |  |  |  |  |  |
| **Total patients** | **11** | | **72** | | | |  | **17** | | | **64** | | |  | | **2** | | | | | | **10** | | | | |  |  | | **1** | | **3** | |  | | **180** | |  |
|  |  | |  | | | |  |  | | | | | |  | |  | | | |  | | | |  | | |  |  | | | | | | | | | |  |
|  |  | |  | | | |  |  | | | | | |  | |  | | | |  | | | |  | | |  |  | | | | | | | | | | |
| **Number of patients with surgical procedures for intracranial hypertension** |  | | | |  |  |  |  | | | | |  |  | | | | |  | |  | | | |  |  | | | | | | | | | | | | |
|  |  | | | |  |  |  |  | | | | |  |  | | | | |  | |  | | | |  |  | | | | | | | | | | | | |
| 0 surgical procedure | 3 (1.6%) | | 57 (31.7%) | | | |  | 4 (2.3%) | | | 43 (23.8%) | | |  | | 1 (0.5%) | | | | | | 2 (1.2%) | | | | |  | 0 (0%) | | | | 1 (0.5%) | |  | | 111 (61.7%) | |  |
|  |  |  |  |  |  |  |  |  |  |  |  |  |  |  |  |  |  |  |  |  |  |  |  |  |  |  |  |  |  |  |  |  |  |  |  |  |  |  |
| 1 surgical procedure | 3 (1.6%) | | 4 (2.3%) | | | |  | 3 (1.6%) | | | 4 (2.3%) | | |  | | 0 (0%) | | | | | | 4 (2.3%) | | | | |  | 1 (0.5%) | | | | 1 (0.5%) | |  | | 20 (11.1%) | |  |
|  |  |  |  |  |  |  |  |  |  |  |  |  |  |  |  |  |  |  |  |  |  |  |  |  |  |  |  |  |  |  |  |  |  |  |  |  |  |  |
| 2 to 4 surgical procedures | 3 (1.6%) | | 11 (6%) | | | |  | 5 (2.8%) | | | 14 (7.9%) | | |  | | 1 (0.5%) | | | | | | 3 (1.6%) | | | | |  | 0 (0%) | | | | 1 (0.5%) | |  | | 38 (21.1%) | |  |
|  |  |  |  |  |  |  |  |  |  |  |  |  |  |  |  |  |  |  |  |  |  |  |  |  |  |  |  |  |  |  |  |  |  |  |  |  |  |  |
| ≥ 5 surgical procedures | 2 (1.2%) | | 0 (0%) | | | |  | 5 (2.8%) | | | 3 (1.6%) | | |  | | 0 (0%) | | | | | | 1 (0.5%) | | | | |  | 0 (0%) | | | | 0 (0%) | |  | | 11 (6.1%) | |  |
|  |  | |  | | | |  |  | | |  | | |  | |  | | | | | |  | | | | |  |  | | | |  | |  | |  | |  |
| **Total patients** | **11** | | **72** | | | |  | **17** | | | **64** | | |  | | **2** | | | | | | **10** | | | | |  | **1** | | | | **3** | |  | | **180** | |  |
