# Supplementary material for: Mortality in Children with Optic Pathway Glioma Treated with Up-Front BB-SFOP Chemotherapy
Source: PLoS One. 2015 Jun 22;10(6):e0127676. doi: 10.1371/journal.pone.0127676 (PMC4476571; doi:10.1371/journal.pone.0127676)
Supplement: S3 Table — (DOC) [file pone.0127676.s005.doc]

**S3 Table. Characteristics of dead patients depending on the time between diagnosis and death: Group 2.**

|  | **Death between 6 and 12 years after diagnosis (group 2)** | | | | | | | | | | | **Total**  **n = 11** |
| --- | --- | --- | --- | --- | --- | --- | --- | --- | --- | --- | --- | --- |
|  |  |  |  |  |  |  |  |  |  |  |  |  |
| **Time between diagnosis and death (years)** | 6.1 | 7.2 | 7.5 | 8 | 8.1 | 9.1 | 10.2 | 10.7 | 10.9 | 11.4 | 11.9 | **Median 10.7** |
|  |  |  |  |  |  |  |  |  |  |  |  |  |
| **Sex** |  |  |  |  |  |  |  |  |  |  |  |  |
| Male | + | + |  |  | + |  |  | + |  |  |  | **4** |
| Female |  |  | + | + |  | + | + |  | + | + | + | **7** |
|  |  |  |  |  |  |  |  |  |  |  |  |  |
| **Age at diagnosis (years)** | 0.4 | 1.2 | 0.7 | 6.8 | 3.8 | 13.6 | 0.8 | 0.6 | 4.9 | 0.7 | 0.7 | **Median 0.8** |
|  |  |  |  |  |  |  |  |  |  |  |  |  |
| **NF1** |  |  |  |  |  |  |  |  |  |  |  |  |
| yes |  |  |  |  | + |  |  |  | + |  |  | **2** |
| no | + | + | + | + |  | + | + | + |  | + | + | **9** |
|  |  |  |  |  |  |  |  |  |  |  |  |  |
| **Intracranial hypertension at diagnosis** |  |  |  |  |  |  |  |  |  |  |  |  |
| yes |  | + |  | + |  |  |  |  |  |  |  | **2** |
| no | + |  | + |  | + | + | + | + | + | + | + | **9** |
|  |  |  |  |  |  |  |  |  |  |  |  |  |
| **Diencephalic syndrome at diagnosis** |  |  |  |  |  |  |  |  |  |  |  |  |
| yes | + | + |  |  |  |  |  |  |  |  | + | **3** |
| no |  |  | + | + | + | + | + | + | + | + |  | **8** |
|  |  |  |  |  |  |  |  |  |  |  |  |  |
| **Radiotherapy** |  |  |  |  |  |  |  |  |  |  |  |  |
| yes |  |  | + | + | + | + |  |  |  | + | + |  |
| no | + | + |  |  |  |  | + | + | + |  |  |  |
|  |  |  |  |  |  |  |  |  |  |  |  |  |
| **Number of lines of chemotherapy after BBFOP** |  |  |  |  |  |  |  |  |  |  |  |  |
| 0 |  |  |  |  |  |  | + |  |  |  |  | **1** |
| 1 |  | + |  | + |  | + |  |  |  |  |  | **3** |
| 2-4 | + |  | + |  | + |  |  | + | + | + | + | **7** |
| ≥ 5 |  |  |  |  |  |  |  |  |  |  |  | **0** |
|  |  |  |  |  |  |  |  |  |  |  |  |  |
| **Cause of death** |  |  |  |  |  |  |  |  |  |  |  |  |
| tumor progression | + | + | + | + | + | + |  | + |  | + | + | **9** |
| vascular problem |  |  |  |  |  |  | + |  |  |  |  | **1** |
| second tumor |  |  |  |  |  |  |  |  | + |  |  | **1** |
| chemotherapy complication |  |  |  |  |  |  |  |  |  |  |  | **0** |
