# Supplementary material for: Mortality in Children with Optic Pathway Glioma Treated with Up-Front BB-SFOP Chemotherapy
Source: PLoS One. 2015 Jun 22;10(6):e0127676. doi: 10.1371/journal.pone.0127676 (PMC4476571; doi:10.1371/journal.pone.0127676)
Supplement: S4 Table — (DOC) [file pone.0127676.s006.doc]

**S4 Table. Characteristics of dead patients depending on the time between diagnosis and death: Group 3.**

|  | **Death more than 12 years after diagnosis (group 3)** | | | | | | | | | | **Total**  **n = 10** |
| --- | --- | --- | --- | --- | --- | --- | --- | --- | --- | --- | --- |
|  |  |  |  |  |  |  |  |  |  |  |  |
| **Time between diagnosis and death (years)** | 12.1 | 13.1 | 13.1 | 13.9 | 14 | 14.4 | 14.7 | 15.5 | 17.2 | 18.1 | **Median 12.6** |
|  |  |  |  |  |  |  |  |  |  |  |  |
| **Sex** |  |  |  |  |  |  |  |  |  |  |  |
| Male | + |  |  |  |  |  | + |  |  | + | **3** |
| Female |  | + | + | + | + | + |  | + | + |  | **7** |
|  |  |  |  |  |  |  |  |  |  |  |  |
| **Age at diagnosis (years)** | 2.4 | 1 | 10.3 | 3.7 | 3.5 | 0.8 | 2.9 | 0.4 | 0.9 | 1.8 | **Median 2.4** |
|  |  |  |  |  |  |  |  |  |  |  |  |
| **NF1** |  |  |  |  |  |  |  |  |  |  |  |
| yes | + |  | + | + |  |  | + |  | + | + | **6** |
| no |  | + |  |  | + | + |  | + |  |  | **4** |
|  |  |  |  |  |  |  |  |  |  |  |  |
| **Intracranial hypertension at diagnosis** |  |  |  |  |  |  |  |  |  |  |  |
| yes |  |  |  |  | + | + | + |  |  |  | **3** |
| no | + | + | + | + |  |  |  | + | + | + | **7** |
|  |  |  |  |  |  |  |  |  |  |  |  |
| **Diencephalic syndrome at diagnosis** |  |  |  |  |  |  |  |  |  |  |  |
| yes |  |  |  |  | + |  |  |  | + |  | **2** |
| no | + | + | + | + |  | + | + | + |  | + | **8** |
|  |  |  |  |  |  |  |  |  |  |  |  |
| **Radiotherapy** |  |  |  |  |  |  |  |  |  |  |  |
| yes |  | + | + |  | + |  |  |  | + |  | **4** |
| no | + |  |  | + |  | + | + | + |  | + | **6** |
|  |  |  |  |  |  |  |  |  |  |  |  |
| **Number of lines of chemotherapy after BBFOP** |  |  |  |  |  |  |  |  |  |  |  |
| 0 |  |  |  | + | + |  | + | + | + |  | **5** |
| 1 |  | + | + |  |  |  |  |  |  | + | **3** |
| 2-4 | + |  |  |  |  | + |  |  |  |  | **2** |
| ≥ 5 |  |  |  |  |  |  |  |  |  |  | **0** |
|  |  |  |  |  |  |  |  |  |  |  |  |
| **Cause of death** |  |  |  |  |  |  |  |  |  |  |  |
| tumor progression | + |  | + |  | + | + | + | + | + |  | **7** |
| vascular problem |  |  |  |  |  |  |  |  |  |  | **0** |
| second tumor |  | + |  | + |  |  |  |  |  | + | **3** |
| chemotherapy complication |  |  |  |  |  |  |  |  |  |  | **0** |
